# Time-Dependent Autonomic Dysregulation and Co-Activation Induced by Periodic Limb Movements in Sleep

**DOI:** 10.3390/jcm14061940

**Published:** 2025-03-13

**Authors:** Marta A. Malkiewicz, Malgorzata Grzywinska, Krzysztof S. Malinowski, Eemil Partinen, Markku Partinen, Jan Pyrzowski, Magdalena Wszedybyl-Winklewska

**Affiliations:** 1 Applied Cognitive Neuroscience Lab, Department of Neurophysiology, Neuropsychology and Neuroinformatics, Medical University of Gdansk, 80-210 Gdansk, Poland; marta.malkiewicz1@wp.pl; 2 Neuroinformatics and Artificial Intelligence Lab, Department of Neurophysiology, Neuropsychology and Neuroinformatics, Medical University of Gdansk, 80-210 Gdansk, Poland; malgorzata.grzywinska@gumed.edu.pl; 3 Department of Neurophysiology, Neuropsychology and Neuroinformatics, Medical University of Gdansk, 80-210 Gdansk, Poland; krzysztof.malinowski@gumed.edu.pl; 4 Helsinki Sleep Clinic, Terveystalo Healthcare, 00100 Helsinki, Finlandmarkpart@me.com (M.P.); 5 Department of Neurology, Helsinki University Central Hospital, 00280 Helsinki, Finland; 6 Sleep Disorder Outpatient Clinic, Department of Psychiatry, Helsinki University Central Hospital, 00280 Helsinki, Finland; 7 Department of Emergency Medicine, Medical University of Gdansk, 80-210 Gdansk, Poland; jan.pyrzowski@gumed.edu.pl

**Keywords:** autonomic co-activation, autonomic nervous system, heart rate variability, periodic limb movements in sleep, PLMS duration, cardiovascular risk

## Abstract

**Background:** Periodic limb movements in sleep (PLMS) are characterised by repetitive, involuntary limb movements that occur during sleep and are often associated with autonomic nervous system dysregulation. While it is known that PLMS influence cardiovascular parameters, the exact role of heart rate variability (HRV) and the balance between sympathetic and parasympathetic activity remains unclear. Previous studies have suggested that longer PLMS events may trigger more pronounced autonomic responses, but the relationship between the duration of PLMS and autonomic dynamics has yet to be fully explored. This study aims to investigate the influence of PLMS duration on autonomic co-activation and its potential cardiovascular implications. **Methods:** A retrospective analysis was conducted on polysomnographic, demographic, and medical data from five patients, encompassing a total of 1348 PLMS events. We measured heart rate (HR), high-frequency HRV (HF-HRV), systolic blood pressure (SBP), and diastolic blood pressure (DBP) for 10 heartbeats before and 10 heartbeats after each PLMS series. A time–frequency approach was used, employing 10 RR interval segments to analyse HF-HRV dynamics. Statistical analysis was performed using IBM SPSS Statistics (v. 28.0.0.0), and the Kruskal–Wallis test was used to assess statistically significant deviations from baseline. **Results:** HF-HRV increased during PLMS, indicating enhanced parasympathetic activation. No significant changes in mean DBP or SBP were observed with leg movements of <2.1 s. However, with movements of >2.1 s, significant increases in DBP and SBP were noted, suggesting sympathetic activation. Longer PLMS events were associated with greater parasympathetic activity, while the absence of HR changes indicates concurrent sympathetic activation, supporting autonomic co-activation. **Conclusions:** Our study indicates that PLMS events lasting >2.1 s are linked to increased parasympathetic activity, likely accompanied by sympathetic activation. This simultaneous activation of both branches of the autonomic nervous system, referred to as autonomic co-activation, could lead to autonomic dysregulation and an increased risk of cardiovascular instability, including potentially life-threatening events.

## 1. Introduction

Periodic limb movements in sleep (PLMS) are involuntary, stereotyped movements of the lower limbs that occur in a periodic pattern during sleep, typically involving dorsiflexion of the ankle, extension of the big toe, and occasional flexion of the knee and hip. They are commonly observed in individuals with restless leg syndrome (RLS) [1]; however, they also occur in sleep disorders such as obstructive sleep apnoea, insomnia, and narcolepsy, as well as in neurological and psychiatric conditions, including neurodegenerative diseases, dementia, schizophrenia, and anxiety disorders [2,3,4,5,6]. Emerging evidence suggests a potential link between PLMS and psychiatric disorders, particularly anxiety and depression [7].

Additionally, PLMS have been identified as an independent risk factor for cardiovascular diseases, such as hypertension and stroke, with studies suggesting a possible link to increased cardiovascular and cerebrovascular risk [8].

As psychiatric and cardiovascular diseases become more prevalent, understanding PLMS and its potential impact on these conditions is increasingly important. Anxiety disorders are estimated to affect millions of people worldwide, while depression impacts more than 264 million individuals [9]. Cardiovascular diseases, including hypertension, heart disease, and stroke, are among the leading causes of death, accounting for a significant number of fatalities each year [9]. This underscores the growing relevance of studying PLMS, particularly in relation to these conditions, as gaining insight into its biological mechanisms could provide valuable clinical information [7].

The autonomic nervous system (ANS) plays a crucial role in regulating physiological responses during PLMS, with shifts in sympathetic and parasympathetic activity occurring in reaction to these movements. Sympathetic activation, commonly associated with PLMS, leads to elevated heart rate (HR) and blood pressure (BP), potentially increasing cardiovascular risk in affected individuals [10,11,12]. At the same time, parasympathetic activation may also occur, reflected by an increase in high-frequency HR variability (HF-HRV), suggesting parasympathetic involvement [13]. This simultaneous activation of both branches of the ANS during PLMS is termed autonomic co-activation. Such co-activation can disrupt the autonomic balance, leading to instability and potential cardiovascular consequences because both branches of the ANS are engaged without a dominant influence from either [13].

PLMS may potentially serve as an independent risk factor for cardiovascular diseases. Although transient elevations in BP and HR during PLMS episodes could contribute to the long-term development of hypertension [14], emerging evidence increasingly supports the notion that PLMS itself may represent an independent cardiovascular risk factor.

The frequency and intensity of PLMS episodes may influence the cardiovascular responses observed, but the role of episode duration remains underexplored.

Previous research has shown that PLMS affect autonomic regulation, particularly circulatory function. Sympathetic activation during PLMS leads to increases in nocturnal BP and HR, with significant rises in both at the onset of PLMS, peaking within seconds after each limb movement [14,15,16].

Our previous study, ‘Effect of series of periodic limb movements in sleep on blood pressure, heart rate, and high-frequency heart rate variability’, identified autonomic co-activation in PLMS, providing a foundation for understanding its cardiovascular effects [13]. The current study builds upon this by focusing on the duration of PLMS episodes. We hypothesise that longer PLMS episodes may have a distinct influence on autonomic regulation, potentially leading to more pronounced parasympathetic activity or greater autonomic conflict. The duration of these episodes could modulate the extent of autonomic instability, contributing to an increased risk of cardiovascular events. This hypothesis explores whether the duration of PLMS affects the balance between sympathetic and parasympathetic activity and whether prolonged or repeated activations lead to more significant autonomic dysregulation.

Our study aimed to identify a marker of autonomic dysfunction specifically in the context of PLMS, as autonomic disturbances during these episodes could be linked to cardiovascular implications across various clinical conditions.

By investigating the time-dependent nature of autonomic co-activation, this study aims to address critical questions: Does the duration of PLMS episodes influence the degree of autonomic conflict? Can longer PLMS episodes amplify autonomic instability, increasing the potential for cardiovascular disturbances even in the absence of overt cardiovascular disease? Understanding the time dependency of autonomic responses during PLMS may provide important insights into the physiological processes underlying the disorder and guide more precise clinical assessments for affected individuals.

## 2. Materials and Methods

We conducted a retrospective analysis of polysomnographic (PSG) data, along with demographic and medical information, from 5 patients who experienced a total of 1348 PLMS events. HR, HF HRV, systolic BP (SBP), and diastolic BP (DBP) were measured for 10 heartbeats prior to each PLMS series and for 10 subsequent heartbeats using beat-to-beat analysis. A time–frequency approach, using short segments of 10 RR intervals, was employed to track changes in the measured parameters over time, providing a dynamic perspective of autonomic activity.

Statistical analysis was performed using IBM SPSS Statistics (version 28.0.0.0), and the Kruskal–Wallis test was used to identify significant deviations from baseline values.

We analysed PSG recordings from five patients (three men and two women), aged 32 to 62 years, with PLMS at the Vitalmed Helsinki Sleep Clinic, Finland. All participants had a confirmed diagnosis of RLS made by an experienced neurologist. It is important to note that all individuals in this study had the non-neurodegenerative form of RLS.

The original studies were approved by the local ethics committee of the Medical University of Gdańsk (approval number NKBBN/388/2022), and all participants provided written informed consent.

Each participant underwent a single-night PSG study. None of the patients were receiving pharmacological treatments that could influence or induce PLMS, such as antipsychotics, sedatives, antidepressants, lithium, β-blockers, or calcium channel blockers. Patients with conditions such as renal disease, diabetes mellitus, depression, anxiety disorders, heart disease, psychotic disorders, or arrhythmias were excluded. Participants with an apnoea–hypopnoea index of ≥5 were also excluded.

Medications for other conditions, including those for hypertension, had been stable for at least 2 weeks prior to the PSG study. Detailed inclusion and exclusion criteria are provided in Table 1.

### 2.1. Materials

All PSG recordings were conducted using a SOMNOscreen plus PSG system (Somnomedics, Randersacker, Germany).

The PSG examination included a comprehensive set of parameters: eight electroencephalographic (EEG) leads, two bilateral electro-oculographic leads, bilateral chin electromyographic (EMG) leads, and surface EMG electrodes placed on the left and right anterior tibialis muscles to monitor PLMS. ECG recordings were obtained using three precordial leads. Respiratory patterns during sleep were assessed using a nasal cannula, thoracic and abdominal effort belts, and a finger pulse oximeter.

The PSG recordings also featured continuous, beat-to-beat BP measurements, which were automatically collected using pulse transit time technology [17]. These non-invasive BP measurements were performed without disturbing the participants’ sleep.

Heart rhythm was assessed by detecting QRS peaks in the ECG recording, from which HR was automatically calculated based on RR intervals. ECG signals were sampled at a high frequency of 4 kHz to ensure precision.

Sleep stages and the duration of each limb movement within PLMS events were analysed, with a total of 1348 PLMS samples included in the study. All measurements were performed non-invasively, ensuring they did not disrupt the participants’ sleep, as no awakenings were observed in the sample group.

PLMS scoring followed the standard criteria outlined by the American Academy of Sleep Medicine (AASM) [18]. Movements were classified as PLMS if they exhibited an increase of at least 8 µV above the baseline EMG level, lasted between 0.5 and 10 s, and were followed by a reduction in EMG activity to less than 2 µV above the baseline. To qualify as PLMS, episodes had to consist of at least four movements occurring at intervals of 5–90 s. Only PLMS events without associated arousals were included in the study, and participants who exhibited PLMS with arousals were excluded.

A PLMS series was defined as a sequence of consecutive movements with intervals of <90 s between events. Movements separated by ≥90 s were considered the start of a new series or classified as isolated movements.

PLMS episodes lasting >10 s were classified as PLMS series. None of the participants exhibited isolated PLMS; all episodes occurred within the series. A PLMS series was considered complete if a limb movement was followed by an interval of <10 s [19]. Limb movements overlapping with respiratory events were excluded. Specifically, movements occurring within 0.5 s before the start of, or up to 0.5 s after the end of a respiratory event were not classified as PLMS. In total, 1348 non-arousal-associated PLMS events were selected for analysis.

### 2.2. Methods

The first step involved identifying suitable ECG segments for further analysis. RR intervals were derived from these segments and used to calculate short-term HF-HRV, which reflects parasympathetic modulation. For each time series, mean HR, SBP, and DBP were also assessed.

**Figure 1:**
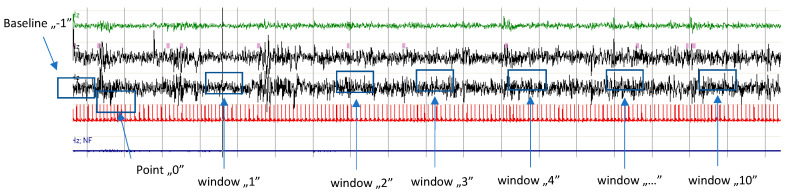
Illustration of PLMS occurring in a series. Point ‘0’ denotes the onset of the first PLMS event in the sequence. Time windows (1–10) correspond to segments of 10 RR intervals following each subsequent PLMS event (1–10). Window “…” illustrates windows 5–9. The baseline is defined as the 10 RR intervals preceding the initiation of the series. For each RR interval within the baseline and time windows, values for HR, SBP, DBP, and HF-HRV were calculated, and the baseline values for HR, SBP, DBP, and HF-HRV were assessed.

Measurements were taken at the following time points: 10 RR intervals immediately before the start of the first PLMS series (baseline; −1), 10 RR intervals at the onset of the first PLMS event in the series (point 0), and a subsequent 10 RR interval segments following each successive PLMS event from 1 to 10 (Figure 1).

HF-HRV was selected for its high sensitivity to parasympathetic modulation, making it ideal for analysing the brief intervals characteristic of PLMS occurrences. This method, which applies consecutive 10 RR interval segments, is consistent with established approaches in time–frequency analysis.

This approach effectively illustrates temporal dynamics in physiological parameters, providing a clear picture of their changes during consecutive PLMS events. To the best of our knowledge, this study is the first to examine dynamic fluctuations in ANS activity, specifically parasympathetic modulation, in relation to PLMS.

HR, HF-HRV, and BP parameters were averaged for each successive PLMS series. To evaluate changes relative to baseline, the values from each PLMS event were expressed as differences from the baseline measurement taken at point ‘−1’.

### 2.3. Statistical Analysis

Statistical analysis was conducted using IBM SPSS Statistics (v. 28.0.0.0). A one-sample test was used to assess whether the measurements exhibited statistically significant differences. Among the parameters, changes in SBP were the only ones that did not reach statistical significance (*p* = 0.0589), whereas other parameters showed significant differences (*p* < 0.05). Normality testing revealed that the data were not normally distributed (*p* < 0.001).

Significant deviations from baseline were assessed using the Kruskal–Wallis statistical test. This test compared the responses of various parameters at different PLMS events against baseline values. HF-HRV values showed a statistically significant rise between the baseline measurement (series ‘−1’) and series ‘9’ (*p* = 0.026).

In this study, the median > 2.1 s approach was employed to evaluate how the duration of PLMS in series affects autonomic activity. The aim was to investigate whether longer phenomena (lasting >2.1 s) have a different impact on sympathetic and parasympathetic co-activation compared to shorter ones.

The analysis focused on differences in the duration of PLMS in series, classified based on the median duration of >2.1 s. Pairwise comparisons were performed as follows:

Comparison between the 1st and 10th movement in the series of PLMS: This comparison was aimed at evaluating the effect of the duration of the first movement (which may reflect the initial phase of the PLMS series) on subsequent movements, particularly the 10th movement, which typically occurs later in the series. We investigated whether differences in the duration of these movements (the first and the last) influenced autonomic activity, particularly with respect to the changing dynamics of sympathetic and parasympathetic activation.

Comparison between the 0th and 10th movement: Because the first movement in the series (the 0th movement) occurs in close proximity to the initial stage of sleep and the 10th movement represents a more advanced phase, this comparison aimed to assess the impact of differences in the duration of these two movements on autonomic activity. The hypothesis was that differences in the duration of these movements could influence autonomic response, possibly leading to changes in sympathetic and parasympathetic balance.

Pairwise comparisons were conducted to determine whether the duration of specific movements significantly influenced autonomic responses, particularly in the context of co-activation of both the sympathetic and parasympathetic systems. Additionally, this analysis aimed to assess how movement duration might affect autonomic balance and whether movements lasting >2.1 s could amplify co-activation, leading to greater autonomic instability.

## 3. Results

The horizontal dashed line represents the baseline mean HF-HRV before the limb movement occurs, serving as a reference point. The vertical dashed line marks the onset of the limb movement, visually distinguishing the pre-movement and post-movement phases. The plot illustrates the evoked changes in HF-HRV, allowing for an analysis of how the autonomic response (in terms of parasympathetic activation) varies during leg movements.

**Figure 2:**
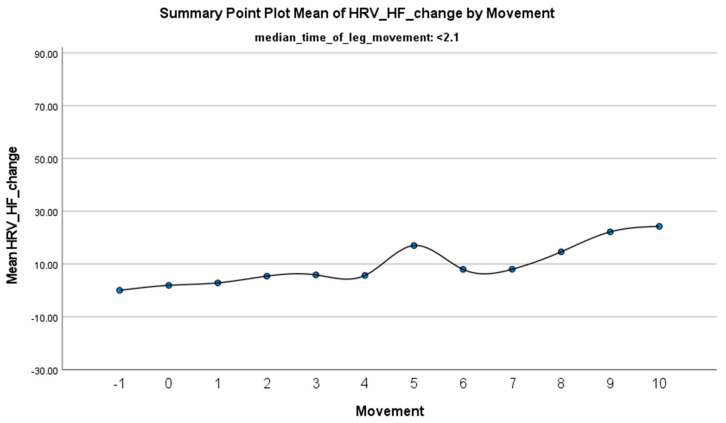
Summary of the mean change in HF-HRV during PLMS with leg movements lasting <2.1 s. The vertical axis represents the mean change in HF-HRV, calculated as the difference between each HRV value and the mean HRV before the limb movement. The horizontal axis indicates the movement series, with ‘−1’ marking the time immediately before the start of the movement and ‘0’ indicating the beginning of the movement. ‘Movement’ on the *x*-axis refers to leg movement.

**Figure 3:**
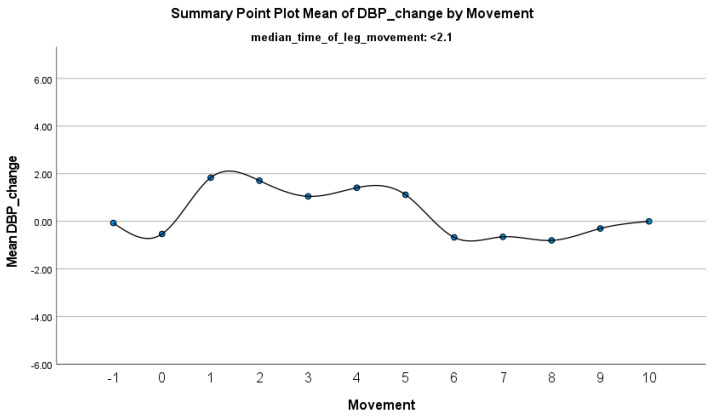
Summary of the mean change in DBP during PLMS with leg movements lasting <2.1 s. The vertical axis represents the mean change in DBP, calculated as the difference between each DBP value and the mean DBP before the limb movement. The horizontal axis indicates the movement series, with ‘−1’ marking the time immediately before the start of the movement and ‘0’ indicating the beginning of the movement. ‘Movement’ on the *x*-axis refers to leg movement.

**Figure 4:**
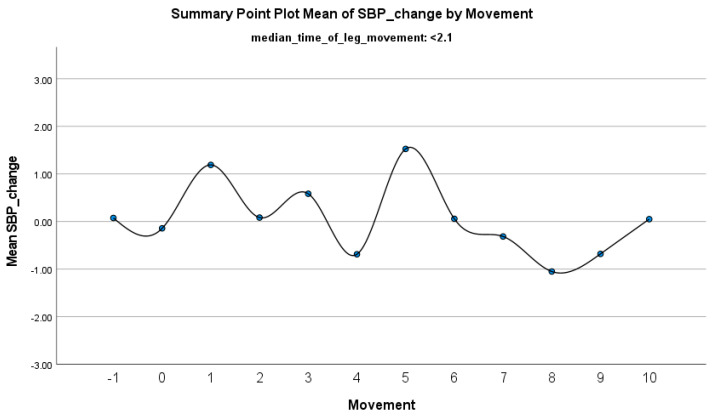
Summary of the mean change in SBP during PLMS with leg movements lasting <2.1 s. The vertical axis represents the mean change in SBP, calculated as the difference between each SBP value and the mean SBP before the limb movement. The horizontal axis indicates the movement series, with ‘−1’ marking the time immediately before the start of the movement and ‘0’ indicating the beginning of the movement. ‘Movement’ on the *x*-axis refers to leg movement.

**Figure 5:**
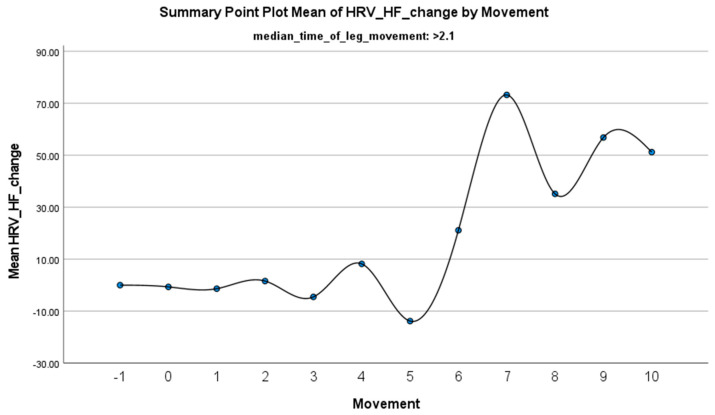
Summary of the mean change in HF-HRV during PLMS with leg movements lasting >2.1 s. The vertical axis represents the mean change in HF-HRV, calculated as the difference between each HRV value and the mean HRV before the limb movement. The horizontal axis indicates the movement series, with ‘−1’ marking the time immediately before the start of the movement and ‘0’ indicating the beginning of the movement. ‘Movement’ on the *x*-axis refers to leg movement.

**Figure 6:**
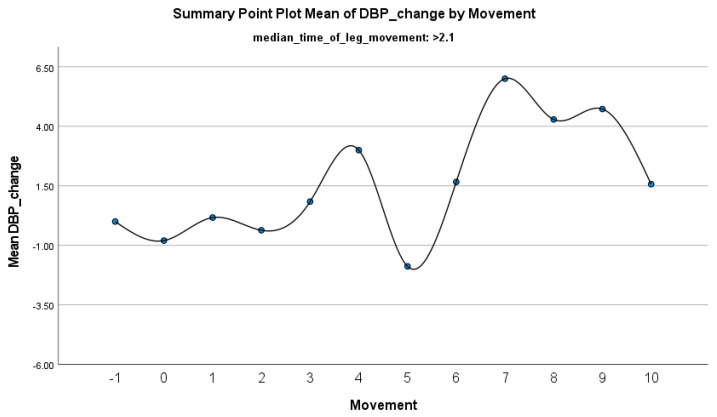
Summary of the mean change in DBP during PLMS with leg movements lasting >2.1 s. The vertical axis represents the mean change in DBP, calculated as the difference between each DBP value and the mean DBP before the limb movement. The horizontal axis indicates the movement series, with ‘−1’ marking the time immediately before the start of the movement and ‘0’ indicating the beginning of the movement. ‘Movement’ on the *x*-axis refers to leg movement.

**Figure 7:**
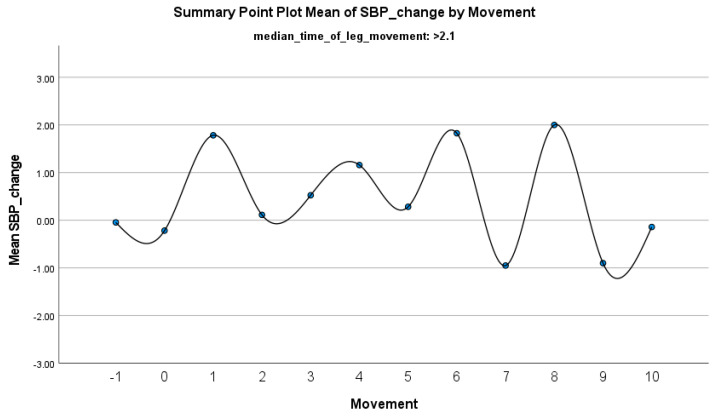
Summary of the mean change in SBP during PLMS with leg movements lasting >2.1 s. The vertical axis represents the mean change in SBP, calculated as the difference between each SBP value and the mean SBP before the limb movement. The horizontal axis indicates the movement series, with ‘−1’ marking the time immediately before the start of the movement and ‘0’ indicating the beginning of the movement. ‘Movement’ on the x-axis refers to leg movement.

The increase in HF-HRV was statistically significant (*p* < 0.01) (Figure 2), indicating enhanced parasympathetic activation during PLMS. However, there was no statistically significant difference (*p* > 0.01) in the mean DBP change when the median time of leg movement was <2.1 s (Table 2), (Figure 3). Similarly, no significant difference (*p* > 0.01) was observed in the mean SBP change under the same condition (Table 2), (Figure 4). By contrast, when the median time of leg movement exceeded 2.1 s, the increase in HF-HRV was also observed (Figure 5), but also in both the mean DBP change (*p* < 0.01) (Table 3), (Figure 6) and the mean SBP change (*p* < 0.01) (Table 3), (Figure 7). This pattern suggests the involvement of sympathetic activation during prolonged episodes, supporting the hypothesis of autonomic co-activation, where both sympathetic and parasympathetic branches of the ANS are simultaneously engaged without clear dominance. This finding is important because it challenges the traditional view of SNS and PNS functioning in opposition. Instead, it highlights a more complex interaction where both branches are activated together, potentially leading to autonomic dysregulation.

## 4. Discussion

Our previous study confirmed autonomic co-activation during PLMS; however, the role of PLMS duration in shaping autonomic responses remained unclear. In this study, we examined how the duration of PLMS episodes influences sympathetic and parasympathetic activation. Our findings suggest that longer PLMS events may amplify parasympathetic activity, potentially contributing to autonomic instability and an increased cardiovascular risk.

Scientific evidence indicates that PLMS are associated with significant increases in HR and SBP. Studies have shown that each individual leg movement can lead to an elevation in HR by approximately 7–10 beats per minute [20] and an increase in SBP by approximately 22 mmHg [15]. Given that the interval between limb movements in PLMS ranges from a few to several dozen seconds, these frequent movements can occur hundreds of times per night, potentially affecting overall cardiovascular function [21].

Our study suggests that the duration of PLMS episodes plays a crucial role in autonomic regulation. Prolonged PLMS episodes exceeding 2.1 s elicit stronger parasympathetic responses, providing insight into the relationship between event duration and autonomic regulation.

While previous studies have primarily focused on sympathetic activation during PLMS, our findings highlight that the duration of movements modulates parasympathetic responses. Specifically, longer episodes may elevate parasympathetic tone to a level that exacerbates autonomic conflict, leading to heightened cardiovascular instability. The simultaneous activation of both the sympathetic and parasympathetic branches of the ANS during prolonged PLMS events disrupts autonomic balance, potentially increasing the risk of cardiovascular events, particularly in individuals with pre-existing conditions.

The complexity of autonomic dynamics during prolonged PLMS episodes underscores the critical need to consider movement duration in cardiovascular assessments. Our findings suggest that analysing the duration of PLMS episodes establishes a new standard for understanding their impact on cardiovascular health. This recognition paves the way for further research into the role of movement duration in autonomic co-activation and its potential long-term implications. Acknowledging the importance of timing in autonomic responses, future studies could refine diagnostic and therapeutic strategies for individuals with PLMS, particularly those with cardiovascular vulnerabilities.

Additionally, studies have shown that PLMS are often followed by increases in EEG activity during sleep, suggesting sympathetic activation. PLMS result from nervous system activation associated with microarousals and increases in HR and BP. A high frequency of PLMS throughout the night could be a potential risk factor for nocturnal arrhythmias and hypertension. They may also lead to sleep fragmentation and sleep deprivation, both of which can have negative health consequences [14].

Winkelman et al. discovered that PLMS are associated with cardiac acceleration even in the absence of arousals [22]. These findings highlight the importance of monitoring cardiovascular parameters in individuals with frequent PLMS because repetitive elevations in HR and SBP may have implications for long-term cardiovascular health.

Further supporting our findings, studies reveal that higher low-frequency HRV (LF-HRV)/HF-HRV ratios during sympathetic activity correlate with increased PLMS frequency, shorter intervals between movements, and longer movement durations, which aligns with previous findings in patients with RLS [23]. The LF-HRV/HF-HRV ratio reflects the balance between sympathetic and parasympathetic activity. LF-HRV captures both influences, while HF-HRV represents parasympathetic activity via the vagus nerve. Higher ratios suggest sympathetic dominance, whereas lower ratios indicate parasympathetic dominance. As LF-HRV is influenced by various factors, the ratio requires cautious interpretation within a broader physiological context [24]. In the same study, Guggisberg et al. observed that sympathetic activation begins before movement onset, suggesting it drives PLMS rather than resulting from it. Furthermore, vagal activity following movements was associated with PLMS magnitude, indicating a connection between the networks generating PLMS and vagal centres [24].

The results reported by Winkelman et al. also support these observations, showing that HR begins to accelerate 2–3 cardiac cycles before a PLM, peaks 4–5 cycles after a PLM, and then falls below pre-movement values 8–10 cycles following a PLM [22]. This biphasic HR response underscores the simultaneous engagement of sympathetic and parasympathetic inputs to the heart.

Previous studies have indicated that cardiac sympathetic activity dominates during PLMS; however, our findings suggest that parasympathetic activity also plays a significant role. This suggests that the sympathetic nervous system does not solely regulate HR during PLMS. Instead, both the sympathetic and parasympathetic systems work in tandem, co-activating the heart in response to PLMS.

Our earlier studies using HRV demonstrated the presence of the HF component (which reflects parasympathetic activity) during PLMS, despite no corresponding increase in HR. The simultaneous increase in HF and the lack of HR elevation suggest the co-activation of both sympathetic and parasympathetic cardiac activity.

In homeostatic mechanisms, situations with a dominant branch of the ANS are typical. However, instances of co-activation of both the sympathetic and parasympathetic systems also occur. PLMS most likely represents such a situation. This simultaneous activation, known as autonomic conflict, may be linked to potentially hazardous cardiovascular events.

The clinical significance of autonomic conflict during PLMS is substantial, particularly in individuals with pre-existing cardiovascular conditions or autonomic dysfunction. Similarly to autonomic stress caused by exposure to cold water, concurrent elevations in parasympathetic and sympathetic activity during PLMS episodes might intensify myocardial electrical irregularities, heightening the risk of arrhythmias.

While our study provides valuable insights, several limitations must be acknowledged. The use of brief time windows limited our ability to assess long-term HRV. Another limitation is that although we adhered to AASM guidelines for scoring PSG recordings and incorporated the International Restless Legs Syndrome Study Group (IRLSSG)/World Association of Sleep Medicine (WASM) guidelines for respiratory event-related leg movements to exclude PLMS episodes potentially influenced by respiratory disturbances, this approach may not fully account for subtle respiratory-related influences that could escape standard scoring criteria. Future studies should explore advanced methods to disentangle respiratory and movement-related autonomic effects.

Moreover, although the sample size was small, no PLMS episodes were associated with arousals, which are linked to increased high-frequency EEG activity and sympathetic activation. Consistent HRV changes across all patients and the analysis of 1348 leg movements strengthen the validity of our findings. However, individuals without arousal-associated PLMS episodes may not fully represent the diversity of PLMS phenotypes. Arousal-associated movements often provoke more pronounced autonomic changes, so studies including these episodes could provide a more comprehensive understanding of PLMS-related autonomic dynamics.

Lastly, the absence of longitudinal follow-up precludes an assessment of the long-term cardiovascular implications of PLMS-induced autonomic co-activation. Prospective studies tracking cardiovascular outcomes over time are needed to clarify the clinical significance of our findings and validate the hypothesis that prolonged PLMS episodes increase cardiovascular risk.

Our findings underscore the complexity of autonomic dynamics during prolonged PLMS episodes and suggest that the duration of PLMS is a critical factor influencing autonomic behaviour. The ability to detect and analyse the duration of PLMS episodes establishes a new standard for understanding how PLMS affect cardiovascular health. This paves the way for further research into the role of movement duration in autonomic co-activation and its potential impact on long-term cardiovascular risk. By recognising the importance of timing in autonomic responses, future research may contribute to the development of improved diagnostic and treatment approaches for patients with PLMS, particularly those with pre-existing cardiovascular risks.

## 5. Clinical Relevance of Autonomic Co-Activation in PLMS

This study highlights two critical findings: the simultaneous activation of the sympathetic and parasympathetic branches of the ANS during PLMS and the significant role of movement duration in modulating these responses. Prolonged PLMS episodes (>2.1 s) amplify autonomic co-activation, potentially disrupting homeostasis and increasing cardiovascular risk, particularly in individuals with pre-existing vulnerabilities.

Notably, parasympathetic activation, indicated by increased HF-HRV components, emerges even without changes in HR or BP. This suggests a nuanced interplay between autonomic branches that is independent of arousal-associated movements, which typically provoke more pronounced sympathetic surges. Such autonomic instability may elevate the risk of arrhythmias and other cardiovascular events.

Additionally, predictive parasympathetic activity—evidenced by elevated HF components preceding PLMS—highlights the importance of timing in ANS responses. These findings align with prior research showing both sympathetic and parasympathetic contributions to autonomic regulation during PLMS.

The method of analysing HRV within short time windows proved effective for capturing dynamic autonomic changes, reinforcing the significance of movement duration in understanding PLMS physiology. Future investigations should prioritise the time-dependent nature of these responses and their implications for cardiovascular health, advancing diagnostic and therapeutic strategies for affected individuals.

Given these findings, evaluating both the frequency and length of PLMS events is critical for identifying individuals prone to autonomic instability. The time-dependent nature of co-activation between the sympathetic and parasympathetic systems may be particularly important in assessing cardiovascular risks. Dysregulation of autonomic HR control in PLMS has been implicated in the development of cardiovascular and cerebrovascular diseases. Understanding these mechanisms and their temporal characteristics could inform clinical strategies aimed at mitigating such risks.

Moreover, the identification of autonomic dysfunction through PLMS could offer valuable insights into the prodromal stages of neurodegenerative diseases, providing an opportunity for early intervention to prevent or slow disease progression. PLMS is frequently observed in conditions such as Parkinson’s disease, Alzheimer’s disease, and other neurodegenerative disorders, where early detection of ANS dysfunction could be pivotal for improving outcomes.

In summary, this study provides new insights into the autonomic changes associated with PLMS, emphasising the importance of both timing and event duration. While the presence of simultaneous sympathetic and parasympathetic activation during PLMS is increasingly recognised, further research is needed to elucidate the precise mechanisms driving these changes. Future studies should explore the impact of PLMS intensity and timing on cardiovascular outcomes, with the goal of improving patient management and long-term health outcomes. By advancing methodologies for HF-HRV analysis and focusing on time-dependent autonomic dynamics, this research lays the groundwork for a more nuanced understanding of PLMS and its implications for overall health.

## Figures and Tables

**Table 1:** Inclusion and exclusion criteria applied in the study.

Inclusion Criteria	Exclusion Criteria
Age of 18–65 years	Age of <18 years
Diagnosis of RLS	Age of >65 years
PLMS without arousal	PLMS with arousal
Treatment for non-excluded medical conditions stable for at least 2 weeks prior to the PSG study	Respiratory events
	Apnoea/hypopnoea index of ≥5
	Use of antipsychotics, sedatives, antidepressants, lithium, β-blockers, or calcium channel blockers

**Table 2:** Presentation of statistical results of the one-sample test for the condition where median_time_of_leg_movement < 2.1 s. Only DBP_change and HF-HRV_change demonstrated statistically significant differences (*p* < 0.05).

One-Sample Test						
	Test Value = 0					
	t	df	Sig. (Two-Tailed)	Mean Difference	95% Confidence Interval of the Difference	
					Lower	Upper
HR_change	−0.251	839	0.802	−0.05357	−0.4722	0.3651
SBP_change	0.510	839	0.610	0.17869	−0.5084	0.8658
DBP_change	3.195	839	0.001	0.49464	0.1907	0.7986
HF-HRV_change	14.411	839	0.000	8.38750	7.2451	9.5299

**Table 3:** Presentation of statistical results of the one-sample test for the condition where median_time_of_leg_movement > 2.1 s. SBP_change, DBP_change, and HF-HRV_change demonstrated statistically significant differences (*p* < 0.05).

One-Sample Test						
	Test Value = 0					
	t	df	Sig. (Two-Tailed)	Mean Difference	95% Confidence Interval of the Difference	
					Lower	Upper
HR_change	−1.600	425	0.110	−0.63427	−1.4136	0.1451
SBP_change	3.961	425	0.000	0.55000	0.2771	0.8229
DBP_change	4.490	425	0.000	1.27512	0.7170	1.8333
HF-HRV_change	7.322	425	0.000	14.26103	10.4325	18.0895

## Data Availability

The data can be made available on request.
